# Supplementary material for: Ultrasound Examination for Cement Extrusion After Uni-Compartmental Knee Replacement
Source: Diagnostics (Basel). 2025 Jan 5;15(1):112. doi: 10.3390/diagnostics15010112 (PMC11720118; doi:10.3390/diagnostics15010112)
Supplement: Supplementary file 1 [file diagnostics-15-00112-s001.zip › Material S1-SWOT analysis.pdf]

SWOT analysis for "Ultrasound Examination for Cement Extrusion After Uni-Compartmental Knee Replacement"

Strengths

This case study introduces ultrasound as a novel tool for evaluating posterior cement extrusion and its effects on soft tissues after knee replacement. It highlights ultrasound's unique ability to provide detailed, non-invasive, radiation-free assessment of soft tissue injuries next to orthopedic implants (metal and cement in this case), which cannot be well delineated by other imaging modalities like computed tomography or magnetic resonance imaging. The study also serves as an educational resource, showing which structures to evaluate in postoperative care.

Weaknesses

The study is based on a single case, limiting its generalizability to broader patient populations. It lacks long-term outcome data and does not compare ultrasound to other imaging modalities, such as magnetic resonance imaging. However, it is well known that despite recent advances, it is inherent weakness of magnetic resonance imaging in delineating soft tissues immediately next to metallic implants, ie. the posterior knee capsule in this case.

Opportunities

The study supports the adoption of ultrasound in postoperative evaluations, particularly when traditional imaging is hindered by artifacts due to surgical implants (metals, cement etc.). It also opens the door for further research to validate ultrasound's role in managing similar complications and for the development of specialized imaging protocols in orthopedics.

Threats

Ultrasound's reliance on operator skill can lead to variability in results. Additionally, resistance from clinicians accustomed to traditional imaging methods and resource limitations in access to advanced ultrasound technology may limit ultrasound's widespread use.
